# Tooth pain accompanying cluster headache in a middle-aged female: A case report

**DOI:** 10.4317/jced.62454

**Published:** 2025-02-01

**Authors:** Sana Ohnuma, Keita Takizawa, Kana Ozasa, Andrew Young, Noboru Noma

**Affiliations:** 1 PhD student, Department of Oral Medicine, Nihon University school of Dentistry Tokyo, Japan; 2 DDS, MSD, Department of Diagnostic Sciences, Arthur Dugoni School of Dentistry, University of the Pacific, San Francisco, United States

## Abstract

Some orofacial pains (OFP) resemble primary headache disorders, but involve the trigeminal nerve V2 or V3 dermatome. The International Classification of Orofacial Pain (ICOP) distinguishes three types of such OFPs: Type 1 (facial pain in addition to headaches), Type 2 (facial pain replacing a previous primary headache), and Type 3 (OFP similar to primary headaches, without a history of headaches). This report describes a 46-year-old female patient with a chief complaint of toothache and severe pain radiating to the left orbital region, and with accompanying autonomic signs and symptoms. The pain persisted despite dental treatments, leading to a diagnosis of cluster headache. The OFP initially matched the Type 1 description, but later changed to match the Type 2 description. This case highlights the need to differentiate nonodontogenic from odontogenic pain for accurate diagnosis and treatment.

** Key words:**cluster headache, autonomic symptoms, Orofacial pain.

## Introduction

The recently-published International Classification of Orofacial Pain (ICOP) categorizes orofacial pains (OFP) into several groups similar to the International Classification of Headache Disorders, Third Edition (1). One category, named “Orofacial pains resembling presentations of primary headaches,” has characteristics akin to primary headache disorders, but involves the trigeminal nerve V2 or V3 dermatomes. The specific conditions are named according to the primary headache that they match, such as “orofacial migraine.” The ICOP also divides this group into three types: Type 1: Headache patients with additional facial pain during the headache attacks. Type 2: Headache patients whose headache attacks have stopped occurring and have been replaced by facial pain attacks of the same characteristics and associated symptoms. Type 3: Headache-naïve patients with de novo orofacial pain attacks that resemble primary headache types (2).

Cluster headache, a typical trigeminal autonomic cephalalgia (TAC) characterized by severe pain and prominent autonomic activation, can radiate to the teeth and maxilla, leading patients to initially consult a dentist. This report describes a case of cluster headache with tooth pain that converted from Type 1 to Type 2.

## Case Report

A 46-year-old female presented to the Oral Medicine Department with the chief complaint of toothache. The pain was severe and radiated to the left orbital region and upper face. It had started two years prior, prompting her to visit a dentist. Despite undergoing pulpectomy, root canal therapy, and tooth extraction in the left maxillary molar region, the pain did not improve. During headache episodes, the pain was accompanied by tearing in the left eye and simultaneous dental pain. The pain was episodic, predominantly occurring in the months of March and April, with 2 to 8 episodes per day, rated at 8 to 9 out of 10 in intensity, lasting from several minutes to several hours. At her initial visit to our department, she reported experiencing the same type of pain localized solely to the left maxilla for the past month.

-Examination findings

**Figure 1:**
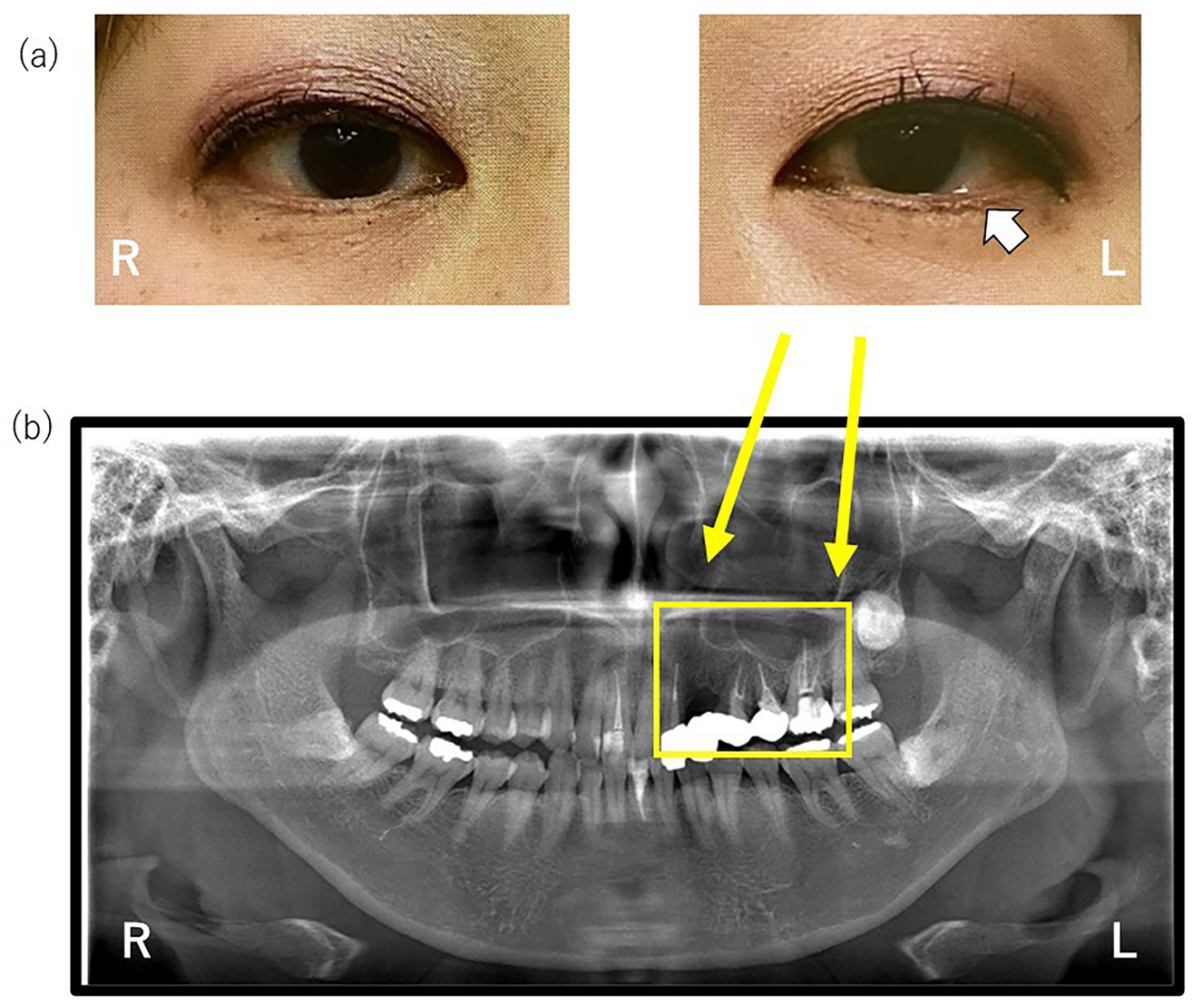
(a) Left ptosis with conjunctival injection was observed. (b) The panoramic radiographs show evidence of tooth extraction and root canal treatment predominantly in the upper left maxillary region. Right (R), Left(L).

Neurological findings were normal, and no pain or tenderness was present in the paranasal sinus region. Examination of the temporomandibular joint (TMJ) in both open and closed positions revealed no abnormalities, but there was tenderness in the left temporalis muscle and bilateral sternocleidomastoid muscles. The eyelids and eyeballs also showed no abnormal findings. Intraoral examination revealed percussion sensitivity in the left maxillary first premolar, but this did not reproduce the chief complaint. Panoramic radiographs showed, in the quadrant of the chief complaint, root canal fillings in the left maxillary lateral incisor, first and second premolars, and first molar, as well as a missing left maxillary canine (Fig. 1).

At the initial visit, tearing associated with the pain could not be observed chairside. The patient was therefore asked to record the duration of the pain attacks, and to take photographs of their facial appearance during those attacks. The patient’s subsequent recordings showed conjunctival hyperemia and mild tearing in the left eye (Fig. 1), and a pain duration of 3 hours. Based on these findings, left-sided cluster headache was suspected, and the patient was referred to a neurology headache clinic. Currently, the patient is being treated at a headache clinic, with a noticeable reduction in headache and orofacial pain.

## Discussion

Cluster headache (3.1), classified under trigeminal autonomic cephalalgias (TACs) in the ICHD-3, is a primary headache disorder characterized by recurrent attacks of severe, strictly unilateral pain (1). The pain typically localizes to the orbital, supraorbital, or temporal regions and lasts 15–180 minutes. Attacks can occur from once every other day to up to eight times daily. Cluster headache is accompanied by ipsilateral autonomic symptoms, including conjunctival injection, lacrimation, nasal congestion, rhinorrhea, miosis, ptosis, eyelid edema, and/or forehead and facial sweating, often with restlessness or agitation.

According to the ICOP classification, cluster headache without head pain is coded as “Orofacial cluster attacks (5.3.1),” a subtype of Trigeminal autonomic orofacial pain (5.3), corresponding to a Type 2 OFP resembling primary headaches (3).

In this case, beginning two years prior, the patient experienced facial and tooth pain during her headache attacks, both of which ceased when each headache attack ceased. This presentation corresponds to Type 1 in the ICOP system. Starting one month prior to her visit to our department, headache no longer occurred during her orofacial pain attacks, though the autonomic signs and symptoms still occurred, corresponding to Type 2 in the ICOP system. This case demonstrates a transition from Type 1 to Type 2 in the same patient.

Benoliel *et al*. further classified cluster headaches into two subtypes based on the pattern of pain radiation (4). Upper Cluster Headache involves pain in the periorbital or ocular regions, forehead, temples, and parietal areas, reflecting the complex neuroanatomy of the trigeminal nerve, particularly the V1 branch (5). Lower Cluster Headache presents with pain in the temporal and suboccipital regions, radiating to the teeth, jaws, neck (5), and cheeks, indicating involvement of the V2 and V3 branches of the trigeminal nerve, and a more complex distribution of pain affecting deeper structures of the head and neck (6,7).

**Figure 2:**
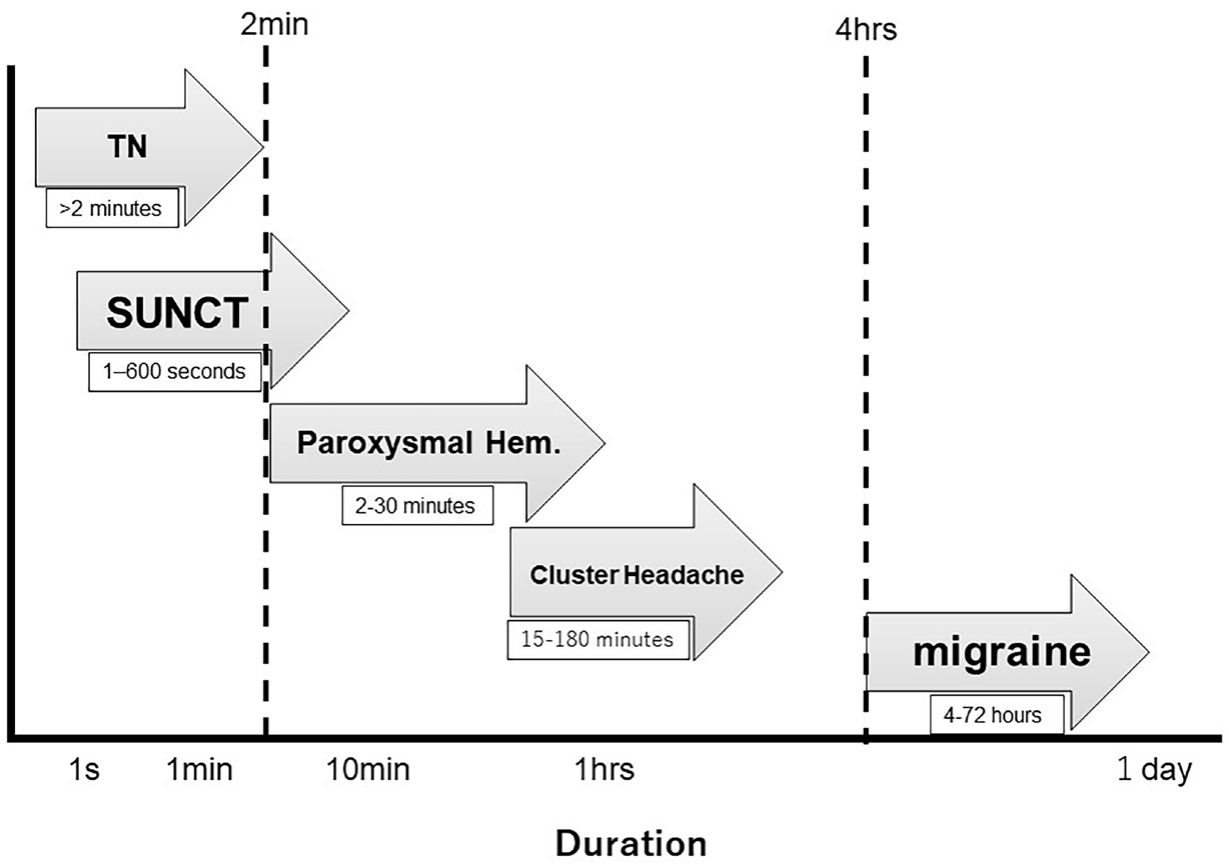
Duration in neurovascular headaches and trigeminal neuralgia. Hem, hemicrania; SUNCT, Short-lasting unilateral neuralgiform headache attacks with conjunctival injection and tearing; TN, trigeminal neuralgia.

Ipsilateral autonomic signs are pathognomonic of TACs, but TAC attacks rarely occur at the time of the consultation, and it is often difficult for patients to recognize their occurrence during a severe pain attack on their own. Yet confirm that a tooth pain is related to a TAC, it is essential to establish that these autonomic signs and symptoms are coinciding with the tooth pain. Cluster headache, paroxysmal hemicrania, and SUNCT syndrome are also clearly defined in terms of pain duration, which aids the diagnostic process (Fig. 2). According to Benoliel *et al*., if a unilateral headache is accompanied by ipsilateral autonomic symptoms and lasts less than 2 minutes, it is very likely to be SUNCT (4). Trigeminal neuralgia (TN) with autonomic symptoms is considered a second differential diagnosis, particularly if a refractory period is present. If a headache with autonomic symptoms persists for more than 4 hours, it is highly likely to be a migraine or a migraine variant with autonomic features, typically observed in the upper third of the head. In this case, a detailed medical interview, along with requesting the patient to measure pain duration and take facial photos (selfies) during the pain attacks, facilitated the diagnosis. Additionally, the panoramic X-ray showed that extractions and root canal treatments were predominantly in the left upper jaw, suggesting that the pain was non-dental in origin and not due to dental causes.

## Conclusions

For dental practitioners, it is important to have knowledge not only of headaches but also of the potential for headaches to cause tooth pain. Recognizing non-dental causes for tooth pain is critical to prevent unnecessary root canals, extractions, and pulp therapy.

## References

[1] (2018). Headache Classification Committee of the International Headache Society (IHS) The International Classification of Headache Disorders, 3rd edition. Cephalalgia.

[2] (2020). International Classification of Orofacial Pain, 1st edition (ICOP). Cephalalgia.

[3] Peng KP, Benoliel R, May A (2022). A review of current perspectives on facial presentations of primary headaches. J Pain Res.

[4] Benoliel R (2012). Trigeminal autonomic cephalgias. Br J Pain.

[5] Cademartiri C, Torelli P, Cologno D, Manzoni GC (2002). Upper and lower cluster headache: clinical and pathogenetic observations in 608 patients. Headache.

[6] van Vliet JA, Eekers PJ, Haan J, Ferrari MD, Dutch RUSSH Study Group (2003). Features involved in the diagnostic delay of cluster headache. J Neurol Neurosurg Psychiatry.

[7] Bahra A, May A, Goadsby PJ (2002). Cluster headache: a prospective clinical study with diagnostic implications. Neurology.

